# CPSP-tools – Exact and complete algorithms for high-throughput 3D lattice protein studies

**DOI:** 10.1186/1471-2105-9-230

**Published:** 2008-05-07

**Authors:** Martin Mann, Sebastian Will, Rolf Backofen

**Affiliations:** 1 Bioinformatics Group, University of Freiburg, Georges-Köhler-Allee 106, 79110 Freiburg, Germany

## Abstract

**Background:**

The principles of protein folding and evolution pose problems of very high inherent complexity. Often these problems are tackled using simplified protein models, e.g. lattice proteins. The CPSP-tools package provides programs to solve exactly and completely the problems typical of studies using 3D lattice protein models. Among the tasks addressed are the prediction of (all) globally optimal and/or suboptimal structures as well as sequence design and neutral network exploration.

**Results:**

In contrast to stochastic approaches, which are not capable of answering many fundamental questions, our methods are based on fast, non-heuristic techniques. The resulting tools are designed for high-throughput studies of 3D-lattice proteins utilising the Hydrophobic-Polar (HP) model. The source bundle is freely available [1].

**Conclusion:**

The CPSP-tools package is the first set of exact and complete methods for extensive, high-throughput studies of non-restricted 3D-lattice protein models. In particular, our package deals with cubic and face centered cubic (FCC) lattices.

## Background

The organisation of bio-molecules, in particular proteins, in the sequence and structure space has recently been attracting increased attention. Particularly questions concerning finding the native structure or investigating the kinetics and evolution of proteins have been widely studied. These problems are often tackled using simplified models such as the Hydrophobic-Polar (HP) model (e.g. Jacob *et al. *[2]). Though abstract, these models are computationally feasible and do allow for deeper insights into fundamental and general principles [2-4].

Several recurring tasks can be identified in such studies using simplified models. Namely, predicting the native structure, classifying whether a sequence is protein-like, calculating its degeneracy and stability, or the design of sequences that optimally fold to a given structure. The problems associated with these tasks are computationally very hard (NP-complete) [5-7]. Nevertheless, these tasks demand for exact and complete (i.e. non-heuristic) methods. It is important to note that stochastic methods cannot be used for proving optimality and in particular proving that a sequence has a unique lowest energy (protein-like) fold [8].

Consequently, with the exception of Yue and Dill [9], all studies requiring complete and exact answers to optimal structure prediction were based on exhaustive enumeration. These studies were, hence, confined to small sequence lengths. In other approaches, structures are artificially restricted to be maximally compact (e.g. filling a 3 × 3 × 3 cube) [10]. This allows for complete enumeration but artificially biases the energy function towards overall hydrophobicity.

Furthermore, many studies are confined to extremely simplified models on the 2D-square or 3D-diamond-lattice [2,11]. The coordination number, a measurement of lattice complexity, is four in both cases. The use of lattices with such a low complexity may lead to oversimplified models that are not able to reproduce real world properties. Park and Levitt [12] have shown that lattices with higher coordination number provide a much better fit to real protein structures. A further hint toward the simplicity of the 2D-lattice is the low computational complexity of inverse folding when compared to the 3D-cubic lattice [7]. The *Constraint-based Protein Structure Prediction (CPSP) *approach by Backofen and Will [13] provides a way to overcome the aforementioned obstacles. The method is tailored to the HP model introduced by Lau and Dill [14]. This model is widely used in the literature [15,16]. CPSP supports complex 3D lattices (currently cubic and face centered cubic) without artificial restrictions (e.g. to be maximally compact). The approach predicts all globally optimal structures together with a proof of optimality. No naive, exhaustive enumeration of all structures is performed and it is as fast as stochastic methods that cannot prove optimality. Backofen and Will [13] showed that the CPSP-approach could fold even sequences of length 200 to optimality within seconds. In contrast, exhaustive structure enumeration as e.g. done by Blackburne and Hirst [17] is restricted to short sequence lengths. For instance, on a 3D-cubic lattice it is only viable to enumerate up to about length 20. In fact, the exact number of structures is only known up to length 23 where there are already more than 5 × 10^15 ^[18]. CPSP uses constraint programming that is commonly applied to hard (NP-complete) problems and, thus, avoids the complete expansion of the whole search space. Hence, constraint-programming techniques are a powerful tool to handle the high complexity that typifies problems related to protein structure. Constraint-programming techniques have successfully been applied to structure prediction with given secondary structure information [19], analysis of NMR data [20], and modeling of protein complexes [21].

Currently, we are not aware of any other complete approach that ensures optimality of the predicted structures in different lattices. There is an alternative to CPSP for the 3D-cubic lattice, the constraint-based hydrophobic core construction method by Yue and Dill [9]. This allows the prediction of optimal structures and proves their optimality. However, using the CPSP-approach, Backofen and Will showed that the method developed by Yue and Dill is not always complete in enumerating all optimal structures [13].

### Complex Lattices

**Figure 1:**
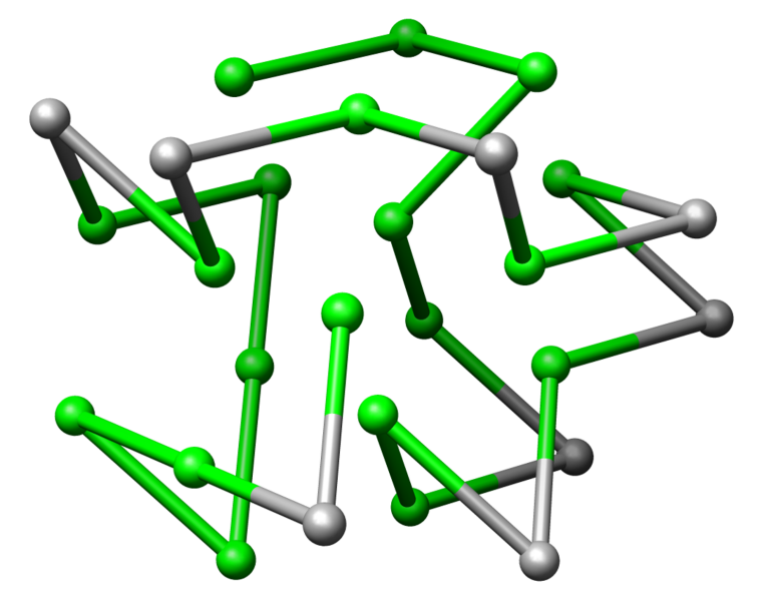
**Structure in FCC lattice model**. One optimal structure of sequence *S*_1 _from Table 2 with 50 HH-contacts in the 3D-face centered cubic (FCC) lattice model. The coloring shows H-monomers in green and P-monomers in grey.

As mentioned before, complete structure enumeration is only applicable to simple, low coordination number lattices. In contrast, the CPSP-approach is built for the more complex 3D-cubic and 3D-face-centered-cubic (FCC) lattices with higher coordination numbers of 6 and 12, respectively. A main feature of the CPSP-tools is their applicability to the unrestricted FCC lattice. The FCC lattice lacks one of the main problems of the 3D-cubic lattice, namely that only sequence positions with different parities form contacts; the parity problem [22]. Modeling protein structures on a FCC lattice, Park and Levitt [12] demonstrated that a good approximation of real protein structures is possible. They achieved a coordinate root mean square deviation of 1.78 Å, whereas a deviation of 2.84 Å was obtained in the 3D-cubic lattice. Recently, Bagci *et al. *[23] have shown that the neighborhood of amino acids in proteins closely resembles a distorted FCC lattice, and that the FCC is best suited for modeling proteins. The CPSP-approach is the first exact method that allows the prediction of provable optimal structures in the FCC lattice. An example is given in Figure 1.

## Implementation

CPSP-tools provides a set of programs that enable typical, modern research tasks to be calculated efficiently and accurately. Here we list the programs each with a typical example application. HPSTRUCT predicts (all) optimal and suboptimal structures as required for investigating properties of low energy conformations, as e.g. studied by Jacob and Unger [16]. The statistical analysis of protein-like sequences, see Blackburne and Hirst [11], requires a degeneracy-based classification of sequences. This is possible with HPDEG. For the exploration of protein evolution, similar to Wroe and Chan [24], one needs to investigate the sequence-structure space. We provide HPDESIGN for sequence design and HPNNET for neutral network computation.

All methods can be applied to HP-sequences in the cubic and the more complex face centered cubic lattice model. Before giving a detailed description of the tools, we first introduce the idea of H-cores, central to these methods.

### H-core database

In the HP lattice models, two monomers form a *contact *if they occupy neighboring positions in the lattice. The *energy *of a structure is defined by the number of contacts between H-monomers, i.e. *HH-contacts*. Thus, an optimal (minimum energy) conformation maximizes the number of HH-contacts. An important observation is that optimal structures show an almost optimal (maximally compact) packing of the H-monomers. Such dispersions of H-monomers without any chain connectivity are called *H-cores*. The compactness of the H-cores is a basic feature that can be used for structure prediction and sequence design. Note that optimal H-cores are independent of a particular sequence and depend only on the number of H-monomers. Hence, compact and nearly compact H-cores can be precalculated and stored in a database. HPSTRUCT and HPDESIGN use this database as a starting point for their calculations (details later). Thereby, redundant computation is avoided, which significantly speeds up the CPSP-approach and related applications.

The enumeration of all optimal H-cores in complex lattice models such as FCC is a computationally hard problem by itself and was solved by Backofen and Will using constraint-programming techniques [25]. Firstly an upper bound on the number of possible contacts for a given number of monomers is calculated via dynamic programming. Subsequently, this information is used to enumerate all compact optimal and almost optimal (*suboptimal*) H-cores for a given number of H-monomers using constraint-programming. Some statistics on the number of H-cores in the 3D-cubic lattice are given in Fig. 4. It shows that the number of H-cores grows exponentially in H-core size but still much slower than the number of structures for a corresponding sequence length.

### HPstruct

#### Motivation

HPSTRUCT implements the CPSP approach, as introduced by Backofen and Will [13], to predict provably optimal structures of 3D lattice proteins in the HP-model. For a given HP-sequence *S *and a given lattice type (cubic or face centered cubic), (all) optimal structures are calculated. The CPSP approach computes the global minimal energy for *S*.

#### Methods

The CPSP-approach is based on the H-core database as described before. For a concrete sequence *S *the approach systematically examines the list of H-cores compatible with *S *in decreasing maximal contact number. For each core, it attempts to thread the sequence through the core. Threading means to find a placement of the monomers of *S *in a self-avoiding walk such that all H-monomers are elements of the given H-core and all P-monomers are outside of the core. Since the H-cores are considered in the order of decreasing contacts, the first successful threading results in a structure with global minimal energy. Note that at this point the algorithm has *proven *that there is no structure of *S *that forms more HH-contacts.

Technically, the threading of a sequence through a core is performed by a constraint program. For this purpose, we formulate the threading problem as a constraint satisfaction problem (CSP) [26]. It constrains the H-monomers of the sequence to the positions in the H-core. Further, it enforces successive monomers along the sequence to be neighbored in the lattice and prohibits the multiple use of a single position. The constraint-programming machinery allows for the enumeration of all valid placements according to the given constraints. In this way, all (sub)optimal structures for a given sequence can be calculated. For a more detailed description of the CSP definition and the mechanisms for solving it see [13].

#### Advanced Features

All resulting structures of HPSTRUCT are returned in absolute move string representation. This compactly encodes the lattice position vectors between successive monomers in the structure and reduces the space consumption for huge data sets.

To handle the common case of highly degenerated sequences (with many optima), HPSTRUCT offers the possibility to limit the number of predicted structures or to generate only a representing subset. Such a subset only contains structures that are separated by at least (a user defined) distance *k*. The distance measure is the hamming distance on the absolute move strings.

### HPdeg

#### Motivation

The degeneracy of an HP-sequence *S *is the number of optimal structures *S *can adopt. It can be calculated using HPDEG and is the base to determine the stability of structures [27]. HPDEG specializes HPSTRUCT and completely counts all optimal structures.

An important application of HPDEG is the classification of sequences as protein-like or not. A sequence is protein-like if it can adopt only one optimal structure (degeneracy 1), a definition applied by Li *et al. *[10] and Huard *et al. *[4] among others.

#### Methods

HPDEG is directly based on the CPSP-approach to compute the degeneracy. Here, all solutions for all arbitrary H-cores/CSPs are calculated. In addition, a significant acceleration of the process can be achieved by the search decomposition methods we introduced in [28]. This is done by identifying sub-chains of the sequence that can be placed independently from each other. Their placements are calculated separately and the resulting numbers are multiplied to the overall structure number of the whole chain. This decomposition strategy results in a speedup of 3-times and higher on average.

### HPdesign

#### Motivation

HPDESIGN solves the inverse folding problem, i.e. the design of sequences that form a given structure *X *as their unique optimum. It allows deeper investigations of sequence-structure relations and a better understanding of general properties of protein folding [29].

The inverse folding problem (IFP) in 3D lattices has been shown by Berman *et al. *[7] to be NP-complete, i.e. it is, as the protein folding problem, a hard computational problem. In contrast, as the same authors show, the IFP in the simple 2D lattice is solvable in polynomial time. This indicates once more the higher complexity of three-dimensional lattice models. To our knowledge, HPDESIGN is the only method applicable to a 3D-model that calculates the desired sequence properties without exhaustive sequence space enumeration.

#### Methods

The approach is based on the CPSP H-core database in order to get a set of good candidate sequences *C*. First, using H-cores ordered by decreasing size and optimality, a matching of the core and the structure is done. For each match a candidate sequence is derived and added to *C*. Afterwards, each *c *∈ *C *is evaluated concerning degeneracy and checked if *X *is its optimal structure.

The candidate set *C*, produced by the filtering step using the H-cores, consists of sequences that can adopt *X *with an optimal or slightly sub-optimal H-core. Therefore, their probability to form *X *as their unique optimum is very high and the size of *C *very small compared to the whole sequence space. The latter is of high importance for the performance of the method.

#### Advanced Features

Often sequences with a special ratio of H/P occurrences or with only limited degeneracy are of interest. Both can be specified using HPDESIGN.

Furthermore, the number of evaluated H-cores is selectable to allow a balancing between runtime and completeness. This is done by adjusting their allowed level of optimality used in the filtering step.

### HPnnet

#### Motivation

The organisation of sequence space in neutral networks provides insights into evolutionary principles [15,30]. Such networks can be expanded using HPNNET. A neutral network for a given structure *X *is an undirected binary graph, where each node represents a sequence that forms *X *as its unique optimal structure. Edges connect evolutionary related sequences, i.e. sequences that differ only in one sequence position, a point mutation. HPNNET expands a neutral network starting from an initial sequence (or a set of sequences) *S *that folds into the structure *X*.

#### Methods

The method follows the generate-and-test paradigm. Recursively, all neighboring sequences of *S *are tested if they adopt *X *as their unique optimum. If so, they are added to the network and their neighbors are checked. Therefore, HPNNET is capable of detecting and expanding connected neutral networks of different structures.

#### Advanced Features

Running HPNNET with *S *as the only start sequence results in the connected component of the network *S *belongs to. However, Blackburne and Hirst [17] have shown by exhaustive enumeration in restricted models that neutral networks may consist of several connected components. To find and study them in complex three-dimensional lattices a combination of HPDESIGN and HPNNET can be used. The independently designed sequences resulting from HPDESIGN have a high chance to belong to different components. HPNNET supports as input such a set of sequences and expands all corresponding connected components. An example is later shown in the results section.

### Utility tools

In addition to those described above, CPSP-tools provides a set of utility programs helpful for lattice protein studies. For instance using HPCONVERT, it is possible to convert between absolute move strings, the 3D-position data in XYZ-, Protein Data Bank (PDB-) and Chemical Markup Language (CML-) format. A move string normalization, as well as a conversion into an orientation independent relative move string, is available for a symmetry independent structure comparison.

HPVIEW interactively visualizes structures in 2D-square, 3D-cubic, and 3D-FCC lattices using the Jmol interface [31].

### Installation and Usage

The package supplies standard installation procedures for Linux based on common tools (GNU automake) and can be compiled and installed easily on current 32- and 64-bit Linux systems (including Cygwin for Microsoft Windows™). The programs are written in C++ for highest performance and provide a slim text-based user interface for efficient pipelining as required for high-throughput experiments. A web front end is under development.

All constraint programming based algorithms utilize the open source Gecode system [32].

The validity of the algorithms has been tested and confirmed on a large set of benchmark problems. The functionality of H-core database access, structure prediction, and degeneracy computation are collected in the C++ CPSP-library. A complete API is included which allows the embedding, extension, and use of the CPSP approach in new programs.

To reduce package size, only a small fraction of the H-core database is included in the source package. This already enables the use of CPSP-tools for short sequences. The complete database is available on request.

## Results and Discussion

For illustration, we provide some scenarios that exemplify the use of CPSP-tools in extending known or enabling new studies. All examples are performed in the unrestricted 3D-cubic lattice with HP-sequences of length 27. Note that for this length there are already more than 10^19 ^possible structures, which makes an exhaustive enumeration inapplicable. Table 1 outlines the performance of programs from CPSP-tools. Table 2 shows the sequences used for Table 1, their optimal energy (*E*), and degeneracy (*deg*). All tasks were performed on an Intel P4 3 GHz (using CPSP-2.0.0).

**Table 1:** Exemplary runs and data. Example runs of the exemplified CPSP-tools application scenarios. The corresponding sequences and structures are given in Table 2. The neutral net *N *is given in Figure 3.

Appl.	Tool	Parameter	Result	Runtime
1	HPDEG	*S*_0_	471354	2.5 s
1	HPDEG	*S*_1_	1	0.2 s
2	HPSTRUCT	*S*_0_	*X*_0_, *E *= -13	0.01 s
2	HPSTRUCT	*S*_1_	*X*_1_, *E *= -22	0.06 s
3	HPNNET	*X*_1_, *S*_1_, *deg *= 1	*S*_1 _.. *S*_4_	9 s
4	HPDESIGN	*X*_1_, *minH *= 17, *so *= 2	*S*_1 _.. *S*_12_	13 m 43 s
4	HPNNET	*X*_1_, *S*_1 _.. *S*_12_, *deg *= 1	*N, S*_1 _.. *S*_14_	1 m

**Table 2:** Data of exemplary runs.

id	Sequence	*E*	*deg*
*S*_0_	PPHPPHHHPHPPPHPHHHPPHPPHHPP	-13	471354
*S*_1_	HHHHHPHHPHPHPHPHPHPHHHHHHPH	-22	1
*S*_2_	HHHHHPHHPHHHPHPHPHPHHHHHHPH	-23	1
*S*_3_	HHHHHPHHPHPHPHPHHHPHHHHHHPH	-23	1
*S*_4_	HHHHHPHHPHHHPHPHHHPHHHHHHPH	-24	1
*S*_5_	HHHHHPHHPHPHHHPHPHHHPHHPHHH	-23	1
*S*_6_	HHHHHPHHPHPHPHPHHHPHPHHPHPH	-22	1
*S*_7_	HHHHHPHHPHPHHHPHHHHHPHHPHHH	-24	1
*S*_8_	HHHHHPHHPPPHPHPHHHPHPHHPHPH	-20	1
*S*_9_	HHHHHPHHPHPHHHPHHHPHPHHPHPH	-22	1
*S*_10_	HHHHHPHHPHPHPHPHHHPHPHHPHHH	-22	1
*S*_11_	HHHHHPHHPHPHHHPHPHPHPHHPHHH	-22	1
*S*_12_	HHHHHPHHPHPHHHPHHHPHPHHPHHH	-23	1
*S*_13_	HHHHHPHHPHPHPHPHPHPHPHHPHPH	-21	1
*S*_14_	HHHHHPHHPHPHPHPHPHPHPHHPHHH	-21	1
*X*_0_	FLUFDDRBLBULFLDRFFUBULDDDR	*S*_0_	
*X*_1_	FLUURDBULLFFRRDDLLBBRULFFR	*S*_1 _.. *S*_14_	

The corresponding sequences and structures for the exemplary runs of CPSP-tools in the 3D-cubic lattice. For each sequence its optimal energy (*E*) and degeneracy (*deg*) is listed. The optimal structures of the sequences are given in absolute move string representation (Forward, Backward, Left, Right, Up and Down). The corresponding neutral net of sequences *S*1 .. *S*14 is given in Figure 3.

**Figure 2:**
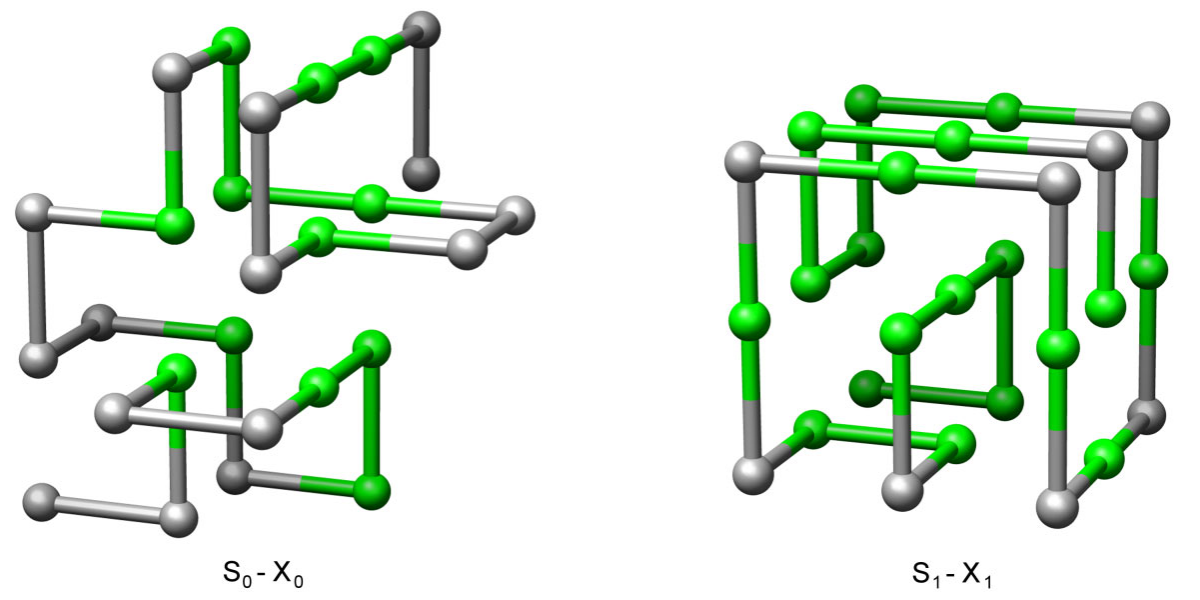
**Structures in 3D-cubic lattice**. An optimal structure *X*_0 _for sequence *S*_0 _and the unique optimal structure *X*_1 _of *S*_1 _from Table 2 in the 3D-cubic lattice. The coloring shows H-monomers in green and P-monomers in grey.

(1) Studies of sequence or structure features of proteins as done by Huard *et al. *[4] require a classification of sequences as protein-like. One way is to classify them by the number of optimal structures, i.e. their degeneracy. The fast calculation of this sequence property by HPDEG allows production of sufficiently large benchmark sets for detailed studies. To illustrate this, we run HPDEG for a random HP-sequence *S*_0 _revealing an enormous degeneracy, which is a frequent finding in the HP-model. As a starting point for the following scenarios, we evaluate the degeneracy of *S*_1_, a sequence with a single optimal structure. The very short runtimes for both checks are given in Table 1.

(2) Calculating the globally optimal structure for a given sequence is the main task in many studies, e.g. see Jacob and Unger [16]. Furthermore, in stochastic folding simulation approaches knowing the minimal possible energy is favorable. Both can be calculated extremely rapidly using HPSTRUCT. Again, We demonstrate this with sequences *S*_0 _and *S*_1_. This results in an energy of -13 and -22 and the optimal structures *X*_0 _and *X*_1_, respectively. Both structures are visualized in Figure 2.

(3) To study protein evolution on the sequence level, neutral networks are widely utilized [17]. Using HPNNET we can span the connected component of the neutral network for a given sequence with a unique optimal structure. Applied to *S*_1 _with *X*_1 _we find four sequences *S*_2 _.. *S*_4 _sharing *X*_1 _as their unique optimal structure. Note, this can be done *without *exhaustive sequence enumeration for a given structure.

(4) The detailed study of neutral networks by Blackburne and Hirst [17] has shown that neutral networks may decompose into connected components. Their results are based on full enumeration of sequences and structures in the diamond lattice. This approach does not extend to complex lattice models due to the enormous size of the structure space as discussed above.

HPDESIGN can overcome that problem by directly designing sequences of the neutral network. Recall that the neutral network contains only sequences with the same unique optimal structure. The described design approach allows one to generate sequences of independent components in the neutral network without exhaustive enumeration. Afterwards, the full components can be expanded via HPNNET.

**Figure 3:**
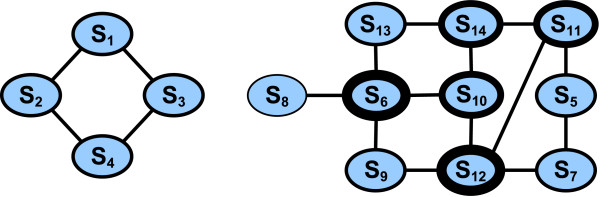
**Neutral net**. Known independent components of the neutral network for structure *X*_1 _from Table 2 in the 3D-cubic lattice. The border size corresponds to the node degree. The structure is visualized in Figure 2.

We apply this approach to the neutral network of the structure *X*_1_. HPDESIGN calculates 12 members of the network (*S*_1 _.. *S*_12_), including the four sequences *S*_1 _.. *S*_4 _known from scenario (3). Expanding the network *N *from these sequences via HPNNET reveals two further sequences *S*_13_, *S*_14 _and two independent connected components as shown in Figure 3.

**Figure 4:**
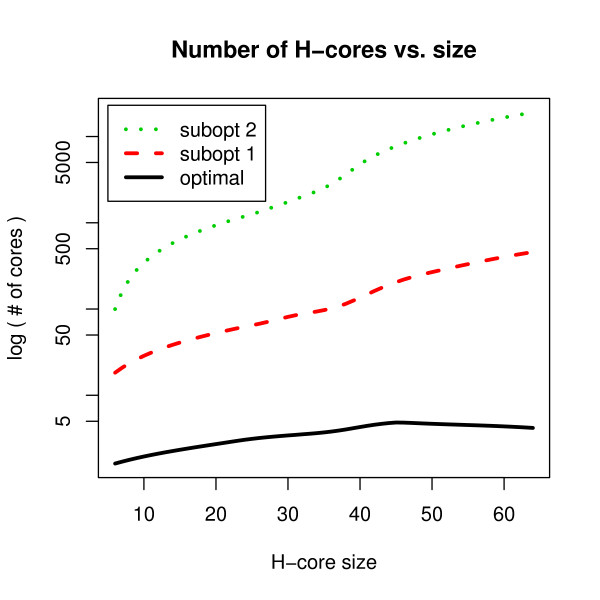
**H-core database statistics**. The number of different H-cores for several number of H-monomers (H-core size) in the 3D-cubic lattice. The three curves represent different levels of optimality of the H-cores.

Preliminary studies performed with CPSP-tools indicate that neutral networks as large as *N *with several large independent components are rare in the unrestricted 3D-cubic model.

## Conclusion

For complex 3D models, mainly heuristic and/or stochastic approaches to search for optimal structures of a given sequence are available [8,33]. However, these methods are (a) incomplete and (b) cannot ensure the global optimality of the predicted structures. In consequence, the investigation of problems requiring this information was only possible using exhaustive enumeration, which is not possible for longer sequence lengths.

The CPSP approach is as fast as common stochastic methods *but ensures *that all predicted structures are globally optimal, and that none are missing. This is done without exhaustive structure space exploration applying constraint-programming techniques. Therefore, it is well suited to many studies in complex 3D models; especially for finding protein-like sequences, the investigation of neutral networks or sequence design. Further applications range from the generation of candidate sets to the validation of results of folding simulations and stochastic optimization methods.

The CPSP-tools package combines several applications in the field of bioinformatics concerning 3D lattice proteins. It allows advanced investigation of problems related to protein structure prediction, sequence evolution, inverse folding, and energy landscapes.

## Availability and requirements

**Project name**: CPSP-tools

**Project home page**: 

**Operating system(s)**: all Linux based systems (including Cygwin for MS Windows™)

**Programming language**: C++

**Other requirements**: Gecode and BIU library (a source bundle is provided)

**License**: BSD-style license

**Any restrictions to use by non-academics**: none

## Authors' contributions

Implementation and software design was done by MM and SW. The CPSP method was developed by RB and SW and extended by SW and MM. The CPSP derived algorithms are designed by all authors. All authors have approved and contributed to the final manuscript.
